# Genome Sequence of the Atypical Symbiotic *Frankia* R43 Strain, a Nitrogen-Fixing and Hydrogen-Producing Actinobacterium

**DOI:** 10.1128/genomeA.01387-15

**Published:** 2015-11-25

**Authors:** Petar Pujic, Alexander Bolotin, Pascale Fournier, Alexei Sorokin, Alla Lapidus, Kerstin H. Richau, Jerome Briolay, Farida Mebarki, Philippe Normand, Anita Sellstedt

**Affiliations:** a Ecologie Microbienne UMR CNRS 5557, Université Lyon I, Université de Lyon, Villeurbanne, France; b INRA, UMR 1319 Micalis-AgroParisTech, AgroParisTech UMR Micalis, Jouy en Josas, France; c Center for Algorithmic Biotechnology, Institute of Translational Biomedicine, St. Petersburg State University, St. Petersburg, Russia; d Department of Plant Physiology, UPSC, Umeå University, Umeå, Sweden; e DTAMB-Biofidal, Département NGS Université Claude Bernard - Lyon 1, Villeurbanne, France

## Abstract

*Frankia* strain R43 is a nitrogen-fixing and hydrogen-producing symbiotic actinobacterium that was isolated from nodules of *Casuarina cunninghamiana* but infects only *Elaeagnaceae*. This communication reports the genome of the strain R43 and provides insights into the microbe genomics and physiological potentials.

## GENOME ANNOUNCEMENT

Nitrogen is an essential element present in the majority of organic molecules in living cells, in particular in amino acids, nucleotides, amino sugars and their polymer-like proteins, nucleic acids, and bacterial envelope constituents. In nature, nitrogen occurs in oxidation states from −3 to +5. Almost all nitrogen compounds can be used and transformed by specific enzymes largely present in microorganisms and, to a lesser extent, in plants and animals. In soils poor in nitrogen, plants depend on its supply by nitrogen-fixing bacteria, either as symbionts associated with plants or as free-living bacteria. Biological nitrogen fixation reduces molecular nitrogen (N_2_) from air using hydrogen (6H++ 6e^−^) and produces two NH_3_ molecules. This reaction is catalyzed by nitrogenases, the metaloenzyme complexes, which have been essential to the sustenance of life on Earth for more than 3.2 billion years (1). *Frankia* are filamentous, Gram-positive, nitrogen-fixing actinobacteria. They infect more than 260 actinorhizal plant species belonging to 8 families of angiosperms (2). The strains were grouped into 4 clusters, cluster no. 1, containing strains infective on *Alnus*, *Myrica*, and *Casuarina*; no. 2 for *Datisca*, *Coriaria*, and *Rosaceae*; no. 3 for *Elaeagnaceae* and *Gymnostoma*; and no. 4 for noninfective (3).

The *Frankia* strain R43, which was originally isolated from *Casuarina cunninghamiana* (4, 5), is a nitrogen-fixing and also hydrogen-producing bacterium (6, 7). It was found not able to reinfect its host *Casuarina*, like other cluster no. 1 strains, but surprisingly, it infected *Elaeagnaceae* belonging to cluster no. 3. For genomic sequencing, its total DNA was prepared from 50 mL of bacterial culture grown in blood agar plate (BAP) medium (8) at 28°C. Cells were harvested, rinsed, and treated with lysozyme, RNase, proteinase K, and phenol-chloroform (50:50 w/w), followed by DNA precipitation using ethanol, and spectrophotometric quantification. The libraries for next-generation sequencing (NGS) and standard sequencing (llumina MiSeq, PacBio, Sanger) were prepared according to manufacturers’ instructions and sequenced with NGS technologies. A genome consensus of 10.45 Mb in 55 contigs was produced using SPAdes assembler (version 3.5.0) in *de novo* hybrid assembly mode (9) exploiting together 6,618,330 paired-end reads of Illumina MiSeq, 65,409 filtered subreads of Pacific Biotech, and the Sanger end reads of 381 fosmids. At 10.45 Mb, this is the largest *Frankia* genome described so far. The consensus sequence produced using Newbler 2.7 independent assembly of 176,528 454 NGS reads and *Frankia* sp. EAN1pec genome sequence (GenBank accession no. CP000820) was exploited as an “untrusted reference” (10). The genome sequence has been annotated using the RAST annotation suite (11). This permitted us to identify loci involved in bacteria-plant symbioses, such as those for nitrogen fixation, uptake hydrogenases, hopanoid biosynthesis, and iron-sulfur cluster biosynthesis that are upregulated in symbiotic *Frankia alni* (8) as well as several genes that are specific to *Frankia* R43 (12).

### Nucleotide sequence accession number.

The draft genome sequence of *Frankia* R43 was deposited at NCBI GenBank under the accession no. LFCW00000000.
